# *In Vitro* Cytotoxicity of Self-Adhesive Dual-Cured Resin Cement Polymerized Beneath Three Different Cusp Inclinations of Zirconia

**DOI:** 10.1155/2019/7404038

**Published:** 2019-05-21

**Authors:** Chang-Yuan Zhang, Yi-Ling Cheng, Xin-Wen Tong, Hao Yu, Hui Cheng

**Affiliations:** ^1^Department of Prosthodontics, School and Hospital of Stomatology, Fujian Medical University, Fuzhou 350002, China; ^2^Institute of Stomatology, Fujian Medical University, Fuzhou 350002, China

## Abstract

The aim of the present study was to evaluate the* in vitro* cytotoxicity of self-adhesive dual-cured resin cement (SADRC) polymerized beneath three different cusp inclinations of zirconia with different light curing time. A commercial SADRC (Multilink Speed) was polymerized beneath zirconia (ZrO_2_) with three different cusp inclinations (0°, 20°, and 30°) for 20 s or 40 s. After being stored in light-proof box for 24 h, the ZrO_2_-SADRC specimens were immersed in DMEM for 72 h and then we got the extract solution, cultured the human gingival fibroblasts (HGF, 8 × 10^3^ per well) with 100% or 50% concentrations of the extract solution for 24 h, 72 h, and 120 h, respectively, and evaluated cytotoxicity of the polymerized SADRC with CCK-8 assay in optical density (OD) values, relative growth rates (RGR), and cytotoxicity grades. Statistical analysis was conducted using a two-way ANOVA followed by* post hoc* Student–Newman–Keuls test. The OD values varied from 0.8930 to 3.2920, the RGR varied from 33.93% to 98.68%, and the cytotoxicity grades varied from 0 to 2. There was significant difference in the OD values among the different cusp inclinations of zirconia (P < 0.001), and there was significant difference in the OD values between the different light curing times in some situations (P < 0.05). The cusp inclination of zirconia affects the* in vitro* cytotoxicity of SADRC. Prolonging the light curing time from 20 s to 40 s can reduce the* in vitro* cytotoxicity of SADRC when the cusp inclination of zirconia is smaller than 20°.

## 1. Introduction

Because of its excellent mechanical and biocompatibility properties [1], yttria stabilized tetragonal zirconia polycrystal (Y-TZP, hereafter simplified to zirconia) has been increasingly used in dentistry [2]. However, due to the lack of glass phase and resistance to aggressive chemical agents, bonding to zirconia still is a challenge for dentists [3], and bonding failure is one of the most common failure modes for zirconia-based restorations. Adhesive resin cements exhibit good biocompatibility, less edge microleakage, good mechanical properties, and excellent esthetic properties and are the most commonly used luting agents for bonding to restoration [4, 5]. But the adhesive cementation process of conventional resin cement is complex and sensitive [6]. Self-adhesive dual-cured resin cement (SADRC), which is easy to use and possesses improved properties, is currently popular luting material in dentistry [7, 8].

The curing kinetics of SADRC are complex [9]. It has been demonstrated that insufficient light curing can result in incomplete polymerization of SADRC [10], and self-curing alone is not sufficient to achieve maximum cement hardening of SADRC [11]. Light curing is important for SADRC [12]. It affects the polymerization efficiency of dual-cured resin cement through high translucent zirconia [13]. In addition, curing time [14], type of light curing unit [15], type of initiator [16], the type [17], and thickness [18] of the restoration can influence the polymerization of dual-cured resin cement.

Moraes et al. founded that self-adhesive resin cement may have a slower polymerization rate and lower polymerization degree than conventional resin cement [19]. The resin cement is not able to achieve 100% polymerization efficiency [20], and the unreacted monomers will be released into proximal tissues [21], such as the pulp and periodontal tissues [22]. The released monomers may cause an inflammatory reaction and other cytotoxicity in the proximal tissues [23]. Unreacted monomers are cytotoxic and genotoxic [24]. It had been founded that released resin monomers cause chemical damage to cells [25]. Trumpaite-Vanagiene et al. studied the cytotoxicity of 3 luting cements and concluded that luting cements may have a cytotoxic potential [26]. The reduction of curing time significantly enhances the cytotoxicity of luting resin cement, and adequate conversion of monomers into polymers can reduce cytotoxicity of resin cement [27]. Therefore, it is of great significance to study the cytotoxicity of the polymerized resin cement.

The artificial restoration surface is irregular, so flat zirconia specimen cannot really reveal the actual situation of clinic. And tooth cusps are the main structures of the restorations surface, so the present study focuses on the* in vitro* cytotoxicity of SADRC polymerized beneath zirconia with three different cusp inclinations. The null hypotheses were as follows: (1) The cusp inclination of zirconia would affect the* in vitro* cytotoxicity of polymerized SADRC and (2) prolonging the light curing time of SADRC would reduce its* in vitro* cytotoxicity.

## 2. Materials and Methods

### 2.1. Monolithic Zirconia Design

**Figure 1:**
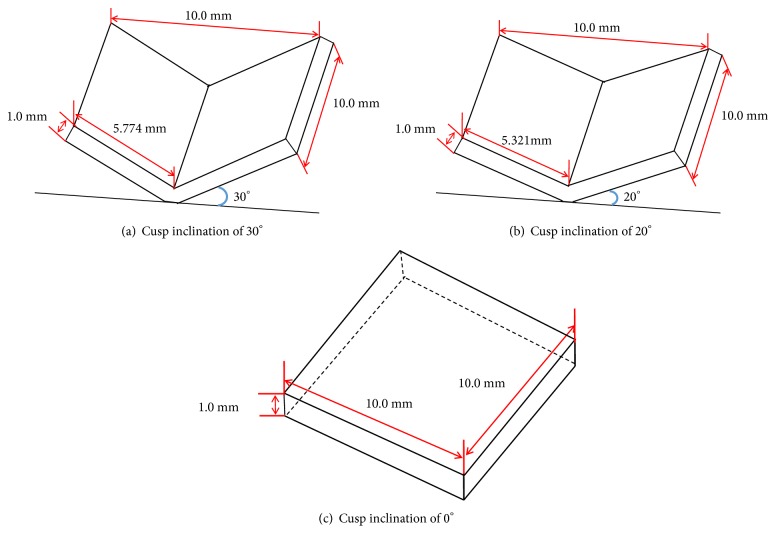
Monolithic zirconia specimens, the width and length of all specimens were 10.0 mm, and the thickness was 1.0 mm.

Three stereolithography (STL) files were drawn with a three dimensional computer-aided design (CAD) software (Solidworks software, Dassault System, France) to simulate three different cusp inclinations (30°, 20°, and 0°) of zirconia, as shown in Figure 1. The length and width of zirconia were 10.0 mm, and the thickness was 1.0 mm.

### 2.2. Monolithic Zirconia Preparation

The three STL files were imported into ZENOTEC CAD software program (Wieland, Germany), and 120 monolithic zirconia specimens were milled (n=40 for each cusp inclination) from zirconia blocks (Wieland, Germany) and sintered in accordance with the manufacturer's instruction. The compositions of the zirconia blocks are shown in Table 1.

### 2.3. Polymerization of Resin Cement

All the bonding surfaces of monolithic zirconia specimens were subjected to 50 *μ*m alumina particle abrasion with a pressure of 1 bar at a distance of 10 mm for 15 s. Then, the monolithic zirconia specimens were ultrasonically cleaned with distilled water for 5 min and air-dried for 5 s.

**Figure 2:**
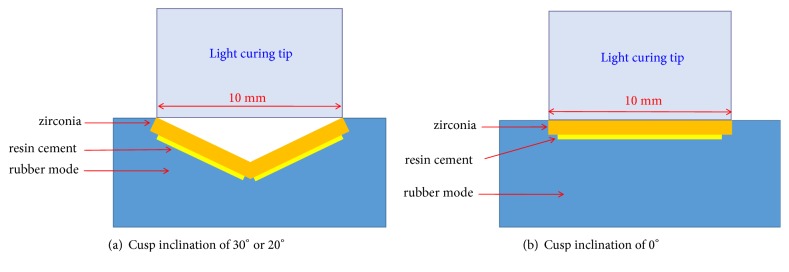
Schematic diagram of light curing process of SADRC beneath zirconia.

A commercial SADRC (Multilink Speed, Ivoclar Vivadent, Liechtenstein. Its compositions were showed in Table 1) was mixed according to the manufacturer's instruction. The mixed SADRC was placed into custom silicon rubber molds and then covered with the previously prepared monolithic zirconia specimens. The SADRC was then light cured through zirconia specimen using a light curing unit (Elipar™ S10, 3M ESPE, USA) with an intensity of 1200 mW/cm^2^ for 20 s or 40 s. The light curing process is shown in Figure 2, and the light curing unit tip was kept close contact to the zirconia surface during the curing process. The output power of the light curing unit was monitored with a radiometer (Bluephase, Ivoclar Vivadent, Liechtenstein) after every five specimens were treated.

### 2.4. Extraction of the Zirconia-Resin Cement Specimens

**Figure 3:**
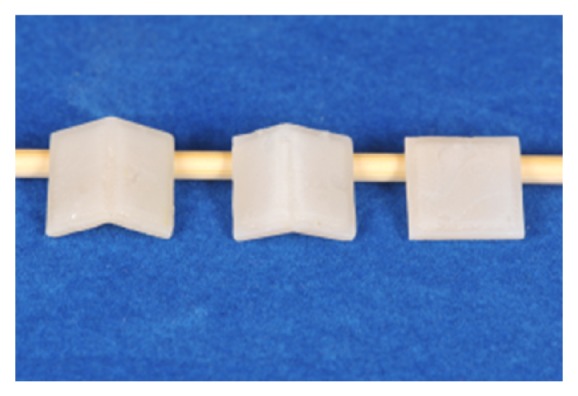
ZrO_2_-SADRC specimens.

After light curing, the zirconia self-adhesive dual-cured resin cement (ZrO_2_-SADRC) and silicon rubber molds were kept in a light-proof box for 24 h [28] at room temperature (22-23°C) [29]. Then the custom silicon rubber molds were gently removed and the ZrO_2_-SADRC specimens were got, as shown in Figure 3. After removal of the trimming of the resin cement, the ZrO_2_-SADRC specimens were disinfected by ultraviolet light for 1 h [30]. Then, the ZrO_2_-SADRC specimens were immersed in Dulbecco's Modified Eagle's Medium (DMEM) (Gibco, Life Technologies, USA) for 72 h at 37°C [31]. The culture medium was supplemented with 10% fetal bovine serum (Gibco, Life Technologies, USA), 100 IU/mL penicillin (Sigma-Aldrich, St. Louis, MO, USA), and 100 *μ*g/mL streptomycin (Sigma-Aldrich, St. Louis, MO, USA). The value of surface area to liquid medium volume was 3 cm^2^ /mL according to the standard [28, 32]. The surface area refers to the surface areas of SADRC in the ZrO_2_-SADRC specimens. Then, the ZrO_2_-SADRC specimens were removed from the DMEM, and the extract solution was diluted with fresh DMEM. The final concentrations of the extract solution were 100% and 50%.

### 2.5. Thawing and Resuscitation of Human Gingival Fibroblast

The 5th passage of human gingival fibroblasts (HGF, ATCC, USA) was trypsinized in cell suspension at a cell density of 0.8 × 10^5^/mL. The sample was seeded in 96-well plates with a cell suspension of 8 × 10^3^ in each well and then cultured at 37°C and 95% humidity in a 5% CO_2_ incubator for 24 h.

### 2.6. Cell Culture Medium Replacement

After the HGF attached and formed a monolayer cell, the cell culture medium was replaced with the extract solution that was extracted from the ZrO_2_-SADRC specimens previously. The experimental groups are shown in Table 2. Repeat eight times for every group. The positive group cell culture medium was replaced with the DMEM containing 30 g/L of phenol. The negative control group was replaced with fresh DMEM. After exposing the cells to the extract solution, all of the 96-well plates were cultured at 37°C, 95% humidity and 5% CO_2_, for 24 h, 72 h, and 120 h.

At the same time, HGF was cultivated in 6-well plates as the experimental groups, with a cell density of 1.5 × 10^5^/mL, and the cell culture medium was replaced with the relevant extract solution. Cells cultured on a 6-well plate were used for morphology observation and photography.

### 2.7. Cytotoxicity Test

A total of 10 *μ*l CCK-8 (CK04, Dojindo, Kumamoto, Japan) liquor was added into each well of the 96-well plates before cell culture termination. 3 h later, the optical density (OD) values was tested with a light absorption enzyme (Bio-Tek ELX808, Bio-Tek, USA) at 450 nm wavelength. The relative growth rates (RGR) of all experimental groups were calculated using the following equation [33]:

  RGR = OD of Experiment group / OD of Negative control group × 100%.

The cytotoxicity in each group was graded as follows [34, 35]:

  Grade 0, noncytotoxic: RGR > 90%.
  Grade 1, slightly cytotoxic: RGR = 60–90%.
  Grade 2, moderately cytotoxic: RGR = 30–59%.
  Grade 3, severely cytotoxic: RGR ≤ 30%.

An inverted phase contrast microscope (DMIL/DFC295, Leica, Germany) was used to observe and photograph the cellular morphology in the 6-well plates.

### 2.8. Statistical Analysis

The OD values were statistically analyzed using a software package (SPSS 20.0, IBM, USA) at a significance level of 0.05. The Shapiro-Wilk test was applied to confirm the normal distribution of the data before ANOVA. The cytotoxicity of SADRC polymerized with different cusp inclinations of zirconia and different light curing time was compared in terms of OD values using two-way ANOVA followed by* post hoc* Student–Newman–Keuls test.

## 3. Results

**Figure 4:**
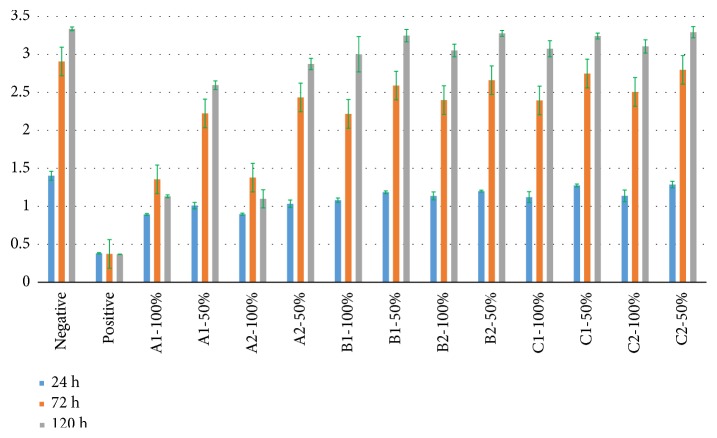
OD values of all experimental groups.

The OD values, RGR, and cytotoxicity grades of all experimental groups are shown in Tables 3, 4, and 5 and Figure 4.

The OD values increased as the culturing time increased, except positive group, A1-100% group, and A2-100% group. The OD values of A1-100% group and A2-100% group increased slightly from 24 h to 72 h and reduced from 72 h to 120 h. All the OD values of experimental groups were lower than that of negative control group for 24 h culturing time. And as the culturing time increased, the difference gradually decreased. For 120 h culturing time, there were no significant differences in OD values between negative control group and B1-50%, B2-100%, B2-50%, C1-100%, C1-50%, C2-100%, and C2-50% groups (P < 0.05).

As the culturing time increased, the RGR of positive group, A1-100% group, and A2-100% group reduced. In contrast, the RGR of other experimental groups increased as the culturing time increased. To the same extract solution, the RGR of 100% concentration groups were lower than that of 50% concentration groups.

**Figure 5:**
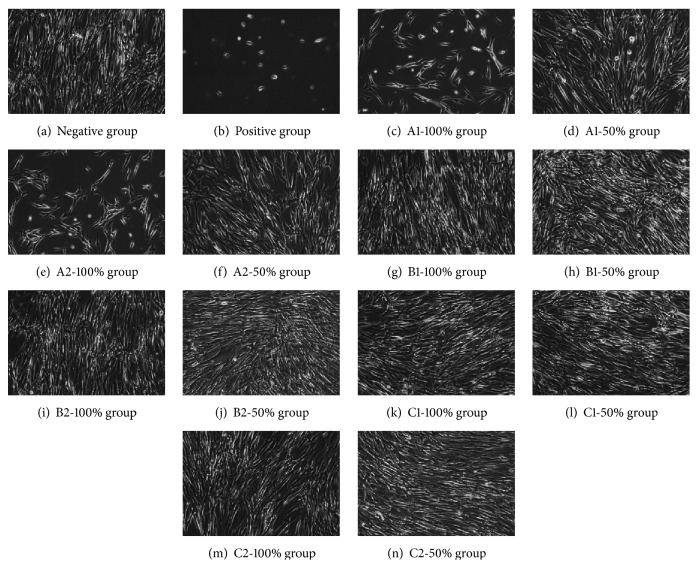
Cellular morphology of all experimental groups with culturing time of 120 h (×100).

The representative cellular morphology of all experimental groups at 120 h culturing time is shown in Figure 5. The cells were mainly fusiform, which is the same as the negative control group. There were no obvious differences in the cell density among B1-100%, B1-50%, B2-100%, B2-50%, C1-100%, C1-50%, C2-100%, and C2-50% and negative control groups. However, the cell density in A1-100% and A2-100% groups was obviously lower than that of other groups, and some of the cells were oval. The cell density of A1-50% and A2-50% groups was lower than other groups but higher than that of A1-100% and A2-100% groups, and some of the cells were oval. There were almost no cells in positive group, the cells were oval, and there were no fusiform cells.

Two-way ANOVA showed that cusp inclination significantly affects the OD values (P < 0.001), regardless of the culturing time or concentration of extract solution. For the culturing time of 24 h, the light curing time did not significantly affect the OD values among the different groups (P = 0.083 for 100% concentration groups, P = 0.098 for 50% concentration groups), but the light curing time significantly affected the OD values among the different groups in the culturing time of 72 h and 120 h (P ≤ 0.001). There was no interaction between cusp inclination and light curing time for 100% concentration of extract solution (P > 0.05), and there was an interaction between cusp inclination and light curing time for 50% concentration extract solution (P < 0.05).

## 4. Discussion

Cytotoxicity assays are the initial screening tests to evaluate the biocompatibility of biomaterials [36]. Cytotoxicity is a biocompatibility test performed on mammalian cells in culture. Extraction, direct contact, and indirect contact are the commonly used methods for cytotoxicity test. Extraction experiment can reflect the cytotoxicity of the tested materials, and the toxic effect through dilution is the main mode of cytotoxicity test for dental materials [37]. So the present study evaluated the* in vitro* cytotoxicity of SADRC polymerized beneath zirconia through extraction. The most common diameter of light curing tip is 10 mm, and 10 mm correlates well with the size of a posterior crown [38]; therefore the zirconia specimens were designed to have a length and width of 10 mm. Furthermore, 30° cusp inclination commonly refers to the tooth cusps of premolars, and the cusp inclination of the molar is close to 20°, while the 0° indicates the labial surface of incisors. So the designed cusp inclinations in present study were 0°, 20°, and 30°. All these designs were attempt to simulate the actual state of the bonding of zirconia-based restorations in clinic.

The cells employed for cytotoxicity include stable cell lines and primary cultured cells. Stable cell lines are readily available and have the advantage of being easy to repeat and reliable in results. While the primary cultured cells can more accurate reflect the cytotoxicity of tested materials, but the individual constitution may affect the results of study. The cells employed in the present study were identical to the cells employed in Trumpaite-Vanagiene R's study [26] and were provided by ATCC of USA. These cells were not only from the same source as the dentin pulp, but they were also standard cell lines. Therefore, these cells were capable of revealing the cytotoxicity of SADRC to gingival and pulp cells.

Based on the results, the hypothesis (1) that “the cusp inclination of zirconia would affect the* in vitro* cytotoxicity of polymerized SADRC” was accepted, and the hypothesis (2) that “prolonging the light curing time of SADRC would reduce its* in vitro* cytotoxicity” was partially accepted.

Several chemical compositions may be released from resin cement in liquid phase solvent [39], and the unreacted monomers are the main materials leading to cytotoxicity [40]. It has been verified [41, 42] that the biocompatibility of zirconia is excellent, so the* in vitro* cytotoxicity of ZrO_2_-SADRC specimens was primarily generated from the resin monomers released from the resin cement. All the resin monomers may lead to cytotoxicity in mammalian fibroblasts [23], resulting in cellular damage [43]. Resin monomers are released when the resin cement polymerizes incompletely, or the polymerizer is degraded by saliva and enzymes. More resin monomers may be released if there is insufficient light curing [44]. The resin monomers of the tested SADRC in the present study are UDMA and TEGDMA. Kurt et al. [23] reported that the UDMA and TEGDMA released from self-adhesive resin cements induced cytotoxicity in cells. The OD values of all experimental groups were lower than that of negative control group and the RGR were lower than 100%, implying that all the extract solution had potential cytotoxicity. These results were in accordance with other previous studies [23, 40, 45]. The cytotoxicity grades in B and C groups were 1 or 0, which means non- or slightly cytotoxic. This result indicates that when the cusp inclination of zirconia was equal to or smaller than 20°, the biocompatibility of the SADRC polymerizer conforms to the ISO standard. The cytotoxicity grade of A1-100% group was 1 or 2, which means it is slightly or moderately cytotoxic. This result implies that the incompletely polymerized SADRC may cause damage to pulp cells and periodontic tissues when the cusp inclination of zirconia reaches 30°.

Most of the resin cement specimens in published articles were polymerized directly [23, 26, 29] or cured beneath flat ceramics. However, in clinic, resin cement is polymerized beneath irregular restorations, so curing the resin cement directly or beneath a flat ceramic cannot real reflect the clinical status. Therefore, SADRC was polymerized beneath three different cusp inclinations of zirconia in present study. For the 100% concentration of extract solution, the OD values and RGR of the A groups were significantly lower than that of B and C groups, and the cytotoxicity grades of A groups were higher than that of B and C groups. These results imply that the cytotoxicity of SADRC light cured through zirconia with 30° cusp inclination was higher than other groups. For the same culturing time, the OD values and RGR of C groups were higher than that of other groups, and the cytotoxicity grade was lower than or equal to that of other groups. Even though the cytotoxicity grade was identical, the OD values and RGR of C groups were higher than that of B groups, although the difference was not statistically significant. These results imply that the cytotoxicity of SADRC light cured through zirconia with 0° cusp inclination was lower than that of other groups. These results indicate that the cusp inclination of zirconia significantly affects the cytotoxicity of ZrO_2_-SADRC. The main possible reason for these results is the distance between the light source and the resin cement. Because the light curing distance affects the degree of conversion of resin cement [46], and the distance in C group is the lowest. The distance is a key factor that affects the conversion degree of resin cement [47], and a lower degree of conversion of resin cement might be correlated with an increased cytotoxicity [48]. The second reason is that the light beam was perpendicular to the SADRC in C groups and angled in the A and B groups, and perpendicular light has been recommended in the literature [38]. Finally, the ceramic thickness may affect the light energy delivery [17]. Because of the existing of cusp inclination, the actual thickness of the light transmitted through zirconia was furthest in A groups, a moderate distance in B groups, and shortest in C groups.

Light curing time is an important factor in the polymerization [49] and* in vitro* cytotoxicity [27] of resin cement. In present study, the light curing time significantly affected the cytotoxicity of ZrO_2_-SADRC in some situations (P < 0.05 for specific culturing time). Therefore, prolonging the light curing time is beneficial to decreasing the cytotoxicity of SADRC.

It is important to state that the present study was an* in vitro* study. In the oral environment, the dentine barrier, saliva buffer system, and other protective systems are capable of resisting to cytotoxicity of resin monomers. Furthermore, the oral environment is much more complex than the setting of present study. Therefore, it is necessary to further simulate the oral environment or conduct* in vivo* studies to reveal the real effect of cusp inclination of zirconia to the cytotoxicity of SADRC.

## 5. Conclusions

Within the limitations of present study, the following conclusions may be drawn:

(1) Cusp inclination of zirconia affects the* in vitro* cytotoxicity of SADRC.

(2) For a zirconia restoration with a thickness of 1.0 mm, when the cusp inclination is smaller than 20°, the cytotoxicity of SADRC conforms to ISO standard, regardless of the light curing time is 20 s or 40 s.

(3) When the cusp inclination of zirconia reaches or exceeds 30°, the cytotoxicity of polymerized SADRC did not conform to ISO standard.

(4) Prolonging the light curing time is beneficial to reducing the* in vitro* cytotoxicity of SADRC.

## 6. Clinical Relevance

Cusp inclination of zirconia is an important factor affecting the cytotoxicity of SADRC, so clinicians should pay attention to the cytotoxicity of SADRC when the cusp inclination of zirconia increases.

## Figures and Tables

**Table 1:** Compositions of main materials used in the study*∗*.

Name	Composition
Zirconia	zirconium oxide (ZrO_2_ +Y_2_O_3_ +HfO_2_) > 99%
	yttrium oxide (Y_2_O_3_): 4.5%~6.0%;
	hafnium oxide (HfO_2_): ≤ 5%
	aluminum oxide + other oxides ≤ 1%

Multlink Speed	Base paste: UDMA, TEGDMA,
	polyethylene glycol dimethacrylate
	Catalyst paste: polyethylene glycol
	dimethacrylate, TEGDMA, methacrylated
	phosphoric acid ester, UDMA
	40 vol% fillers, Barium glass Ytterbium trifluoride

*∗*Information provided by the manufacturer.

**Table 2:** Experimental groups of the study.

Groups	Cusp inclination of zirconia	Light curing time	Concentration of extract solution
A1-100%	30°	20 s	100%
A1-50%	30°	20 s	50%
A2-100%	30°	40 s	100%
A2-50%	30°	40 s	50%
B1-100%	20°	20 s	100%
B1-50%	20°	20 s	50%
B2-100%	20°	40 s	100%
B2-50%	20°	40 s	50%
C1-100%	0°	20 s	100%
C1-50%	0°	20 s	50%
C2-100%	0°	40 s	100%
C2-50%	0°	40 s	50%

**Table 3:** Mean and standard deviations OD values of all experimental groups.

Groups	24 h	72 h	120 h
Negative	1.4025 ± 0.0588	2.9057 ± 0.0635	3.3360 ± 0.0245
Positive	0.3812 ± 0.0092	0.3728 ± 0.0073	0.3682 ± 0.0022
A1-100%	0.8930 ± 0.0126	1.3550 ± 0.0299	1.1319 ± 0.0181
A1-50%	1.0092 ± 0.0424	2.2227 ± 0.1077	2.5947 ± 0.0569
A2-100%	0.8964 ± 0.0141	1.3778 ± 0.0534	1.0985 ± 0.1196
A2-50%	1.0331 ± 0.0491	2.4334 ± 0.0626	2.8725 ± 0.0749
B1-100%	1.0806 ± 0.0285	2.2167 ± 0.2035	3.0018 ± 0.2324
B1-50%	1.1852 ± 0.0178	2.5899 ± 0.1059	3.2468 ± 0.0810
B2-100%	1.1367 ± 0.0528	2.3978 ± 0.0723	3.0514 ± 0.0840
B2-50%	1.1999 ± 0.0124	2.6595 ± 0.0886	3.2766 ± 0.0379
C1-100%	1.1207 ± 0.0714	2.3945 ± 0.1058	3.0745 ± 0.1071
C1-50%	1.2744 ± 0.0190	2.7486 ± 0.0901	3.2417 ± 0.0385
C2-100%	1.1371 ± 0.0774	2.5061 ± 0.0745	3.1051 ± 0.0874
C2-50%	1.2856 ± 0.0430	2.7970 ± 0.0958	3.2920 ± 0.0742

**Table 4:** Relative growth rate of all experimental groups.

Groups	24 h	72 h	120 h
Negative	100.00%	100.00%	100.00%
Positive	27.18%	12.83%	11.04%
A1-100%	63.67%	46.63%	33.93%
A1-50%	71.96%	76.49%	77.78%
A2-100%	63.92%	47.42%	32.93%
A2-50%	73.66%	83.75%	86.11%
B1-100%	77.04%	76.29%	89.98%
B1-50%	84.51%	89.13%	97.33%
B2-100%	81.11%	82.52%	91.47%
B2-50%	85.55%	91.53%	98.22%
C1-100%	79.91%	82.41%	92.16%
C1-50%	90.87%	94.59%	97.17%
C2-100%	81.08%	86.25%	93.08%
C2-50%	91.66%	96.26%	98.68%

**Table 5:** Cytotoxicity grades of all experimental groups.

Groups	24 h	72 h	120 h
Negative	0	0	0
Positive	3	3	3
A1-100%	1	2	2
A1-50%	1	1	1
A2-100%	1	2	2
A2-50%	1	1	1
B1-100%	1	1	1
B1-50%	1	1	0
B2-100%	1	1	0
B2-50%	1	0	0
C1-100%	1	1	0
C1-50%	0	0	0
C2-100%	1	1	0
C2-50%	0	0	0

## Data Availability

The data used to support the findings of this study are available from the corresponding author upon request.
